# The Rise of Ophthalmology as a Specialty: Albrecht von Graefe's Pioneering Contributions

**DOI:** 10.7759/cureus.67973

**Published:** 2024-08-27

**Authors:** Joobin Khadamy

**Affiliations:** 1 Ophthalmology, Skellefteå Eye Clinic, Skellefteå, SWE; 2 Ophthalmology, University Hospital of Umeå, Umeå, SWE

**Keywords:** ophthalmic society founding, specialized surgical instruments, eye care history, glaucoma treatment, surgical advancements, ophthalmological specialty, ophthalmoscope use, iridectomy innovation, modern ophthalmology, albrecht von graefe

## Abstract

Albrecht von Graefe (1828-1870) is celebrated as a pivotal figure in the evolution of ophthalmology. He is renowned for his transformative contributions that established the field as a distinct medical specialty separate from general surgery. This biography review delves into von Graefe's pioneering efforts that reshaped ophthalmology, highlighting his innovations and establishing fundamental practices that define modern ophthalmic care. von Graefe's introduction of iridectomy for glaucoma, development of specialized surgical instruments, and advocacy for the use of the ophthalmoscope marked significant advancements in treating eye disorders. Additionally, his role in founding the first ophthalmological society and journal underscored his commitment to creating a structured, scientific approach to the field. Through meticulous examination of his work and its impact, this article underscores how von Graefe's pioneering spirit and dedication fostered the growth of ophthalmology into an independent and respected medical specialty.

## Introduction and background

Ophthalmology traces its origins to ancient civilizations, where early physicians began studying and treating eye diseases. In Egypt, eye care was integral to medicine, as evidenced by the Ebers Papyrus (circa 1550 BCE), which detailed treatments for eye conditions using herbal remedies [1]. The spiritual significance of the eye was also prominent, symbolized by the Eye of Horus. In India, the Sushruta Samhita (around 600 BCE) provided detailed descriptions of eye anatomy and early cataract surgery techniques like "couching" [2]. Greek and Roman advancements, particularly those by Hippocrates and Galen, further deepened the understanding of eye anatomy and its connection to overall health, influencing medical thought for centuries [3].

Despite early advancements in understanding the eye, much of antiquity and the Middle Ages were marked by limited knowledge and rudimentary treatments, often influenced by mystical beliefs. These early practices, however, laid the groundwork for future developments. Georg Bartisch's Ophthalmodouleia (1583) is considered the first major ophthalmology book, providing detailed insights into eye anatomy and surgery [4]. 

In the 18th century, ophthalmology made notable advances, particularly in cataract surgery and scientific knowledge. However, the field lacked the structure of a specialized discipline. By the early 19th century, ophthalmology was still in its formative stages, struggling to define itself as a separate specialty. Figures such as Franz R. Reisinger and Jan Evangelista Purkyně, alongside von Graefe, played crucial roles in shaping modern ophthalmology [5].

The establishment of ophthalmology as a distinct and advanced medical specialty is deeply connected to the pioneering work of Albrecht von Graefe. Over 170 years, "Graefe's Archive for Ophthalmology" has chronicled the growth of the field, with von Graefe's contributions serving as a cornerstone in its development. This review explores von Graefe's transformative impact, his influential private clinic, and the enduring legacy of his work [6].

## Review

Personal life and professional challenges

**Figure 1:**
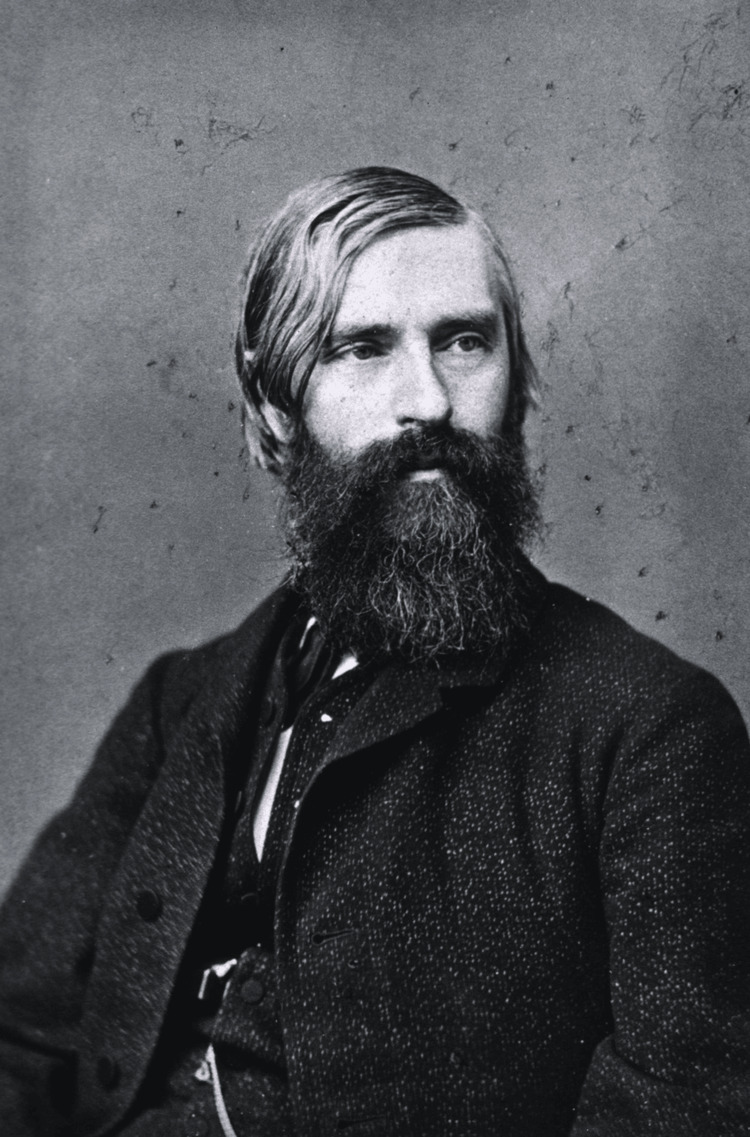
Albrecht von Graefe Reference: [9]

Albrecht von Graefe (Figure 1) was born on May 22, 1828, in Berlin, Germany, into a family with a strong medical background. His father, Karl Ferdinand von Graefe, was a prominent surgeon. Despite his early immersion in the medical world, Albrecht's life was not without personal challenges. He married Anna Adelaide Pauline von Knuth, but their marriage was marred by the stresses of his demanding career and his declining health. They had three children, two of whom died in infancy. von Graefe was a dedicated physician, often working long hours at his private clinic, which took a toll on his personal life and well-being. He suffered from tuberculosis, a condition that would eventually claim his life at the young age of 42. Despite his illness, von Graefe remained deeply committed to his work, continuing to innovate and contribute to ophthalmology until he died in 1870. His dedication to both his family and his profession left a lasting influence on the medical world [7]. His letters and correspondence offer valuable insights into his perseverance and commitment to both his family and his field [8].

During Albrecht von Graefe's time, the field of ophthalmology faced significant limitations due to the low level of medical knowledge and technology. The understanding of ocular diseases and the anatomy of the eye was rudimentary compared to modern standards. Many treatments were based on trial and error, often influenced by outdated theories and misconceptions. Diagnostic tools were primitive, and there was a limited understanding of the underlying pathophysiology of various eye conditions. von Graefe's pioneering work, including his development of new surgical techniques and diagnostic instruments, was conducted in an environment where such innovations were essential for advancing the field. His efforts helped to bridge the gap between early rudimentary practices and the more scientific approach that would come to define modern ophthalmology.

von Graefe's pioneering contributions

Albrecht von Graefe is widely regarded as the father of modern ophthalmology and glaucoma. A child prodigy, he completed medical school by the age of 19. His extensive training under leading ophthalmologists in Prague, Paris, Vienna, and London, as well as his education with Sir William Wilde in Dublin, significantly influenced his approach to eye care. von Graefe's contributions were instrumental in establishing ophthalmology as a separate and advanced medical specialty. von Graefe's research can be categorized into three main phases: his initial studies on conjunctival diseases, sensory physiology, and strabismus; his subsequent work on glaucoma, which included the introduction of peripheral iridectomy in 1857; and his later research focused on advancements in cataract extraction [7].

Iridectomy and Glaucoma Treatment

von Graefe introduced iridectomy as a surgical procedure to treat acute glaucoma, a groundbreaking advancement that significantly improved treatment outcomes [10].

Development of Diagnostic Tools

von Graefe developed the first tonometer for measuring intraocular pressure and pioneered visual field testing, laying the foundation for modern diagnostic practices in ophthalmology [11,12].

Ophthalmoscope and Retinal Examination

By utilizing Hermann von Helmholtz's ophthalmoscope, von Graefe advanced the examination of the posterior eye, enhancing the understanding of conditions such as retinal detachment and choroiditis [13].

**Figure 2:**
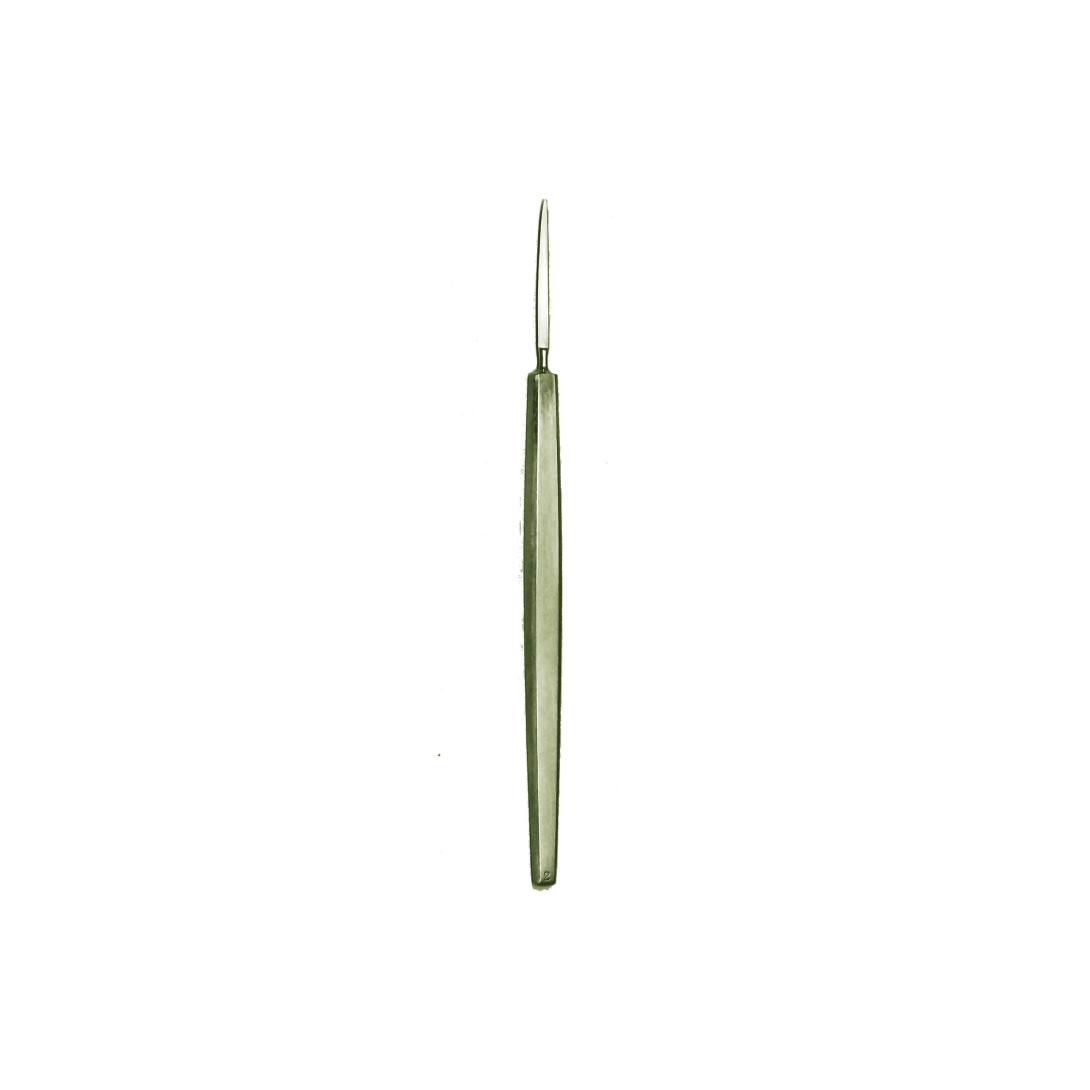
The von Graefe knife Reference: [15]

Cataract Surgery

von Graefe's invention of the "von Graefe knife" (Figure 2) for cataract surgery and his advancements in lens extraction techniques marked significant improvements in surgical precision and patient outcomes [14].

von Graefe's private clinic and influences

von Graefe's private eye clinic, established in Berlin in 1852, became a renowned center for ophthalmic research and education. The clinic treated over 10,000 patients, including notable figures such as the following: (a) Alexandra Feodorovna, the Russian Empress, who was treated for chronic choroiditis; (b) Prince Frederick of Württemberg andPrince William of Württemberg, who underwent significant surgeries; (c) Countess Johanna Bismarck, who was examined in her home; and (d) Countess Anna Adelaide Pauline von Knuth, who later became von Graefe's fiancée [16,17].

The clinic was committed to offering free treatments for the underprivileged, ensuring that even the most disadvantaged individuals had access to essential care.

The clinic attracted numerous international students and assistants, fostering a new generation of ophthalmologists. von Graefe's mentorship and innovations profoundly influenced several prominent ophthalmologists, including the following: (a) Johann Friedrich Horner, known for Horner's syndrome, who was significantly impacted by von Graefe's scientific approach [18]; (b) Richard Liebreich, a prominent figure in ophthalmology who benefited from von Graefe's advancements [19]; (c) Michele Del Monte, influenced by von Graefe's work in cataract surgery; and (d) Julius Jacobson, whose contributions were shaped by von Graefe's pioneering research [20].

Achievements and contributions

Albrecht von Graefe played a pivotal role in establishing ophthalmology as an independent specialty. Before his contributions, eye care was often relegated to general surgeons or was intertwined with other medical practices, lacking a distinct identity. von Graefe's dedication to ophthalmology was evident through his establishment of a private clinic in Berlin, which became a hub for specialized research and training. He was instrumental in founding the German Ophthalmological Society (DOG) in 1857, which provided a formal structure for the field and fostered a collaborative environment for advancing ophthalmic science. Additionally, the founding of "Graefe's Archive for Ophthalmology" in 1854 marked the beginning of a dedicated scientific journal that disseminated new research and innovations. By emphasizing the need for specialized knowledge and techniques, von Graefe helped to elevate ophthalmology from a subspecialty of general surgery to a distinct and respected medical discipline. His work laid the groundwork for modern ophthalmology and ensured its recognition as a crucial and independent area of medical practice. Table 1 summarizes his key achievements and contributions [7]. 

**Table 1:** Key milestones in Albrecht von Graefe's life DOG: German Ophthalmological Society; CRAO: central retinal artery occlusion; CSCR: central serous chorioretinopathy

Year	Event/achievement/contribution
1828	Born on May 22 in Berlin, Germany.
1843-1848	Attended medical school at the University of Berlin.
1852	Founded a leading private eye clinic at Karlstrasse 46, Berlin.
1854	Founded the "Archiv für Ophthalmologie," later named in his honor.
1854	Second to describe retinal detachment, after Ernst Adolf Coccius.
1856	Introduced perimetry for visual field testing.
1856-1857	Performed iridectomy as a treatment for acute glaucoma.
1857	Became the first associate professor of ophthalmology in Germany.
1857	Founded the DOG.
1859	Documented CRAO.
1860	Named first chairman of the Berlin Medical Society.
1860	Demonstrated that blindness linked to cerebral disorders often results from optic neuritis.
1862	Invented one of the first tonometers to measure intraocular pressure.
1863	First to execute intravitreal manipulations.
1865	Invented the "von Graefe knife" for cataract surgery.
1866	Described CSCR.
1867	Developed a new method for cataract treatment through lens extraction.
1868	Appointed head of the eye clinic at Berlin's Charité Hospital.
1866-1870	Served as a full professor of ophthalmology at Charité Hospital and the University of Berlin.
1870	Elected a foreign member of the Royal Swedish Academy of Sciences.
1870	Died on July 20 in Berlin, Germany.

**Figure 3:**
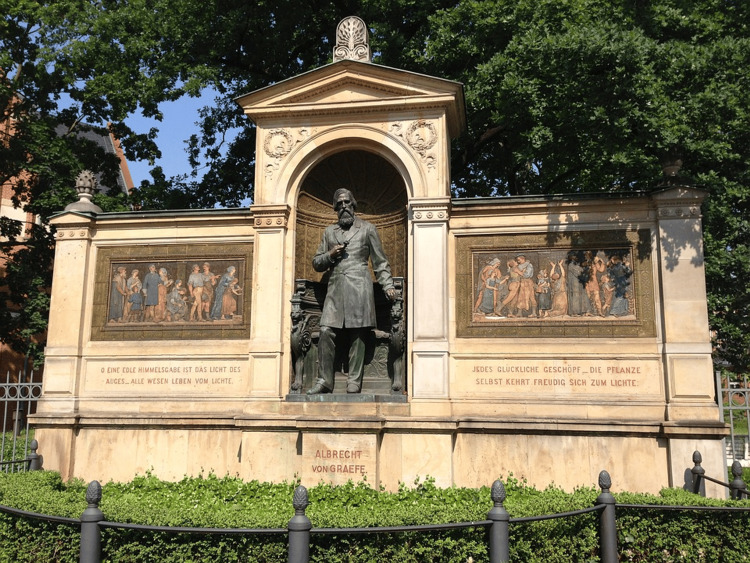
Albrecht von Graefe monument, Berlin, Germany Reference: [21]

Legacy

von Graefe's influence extended beyond his lifetime. Monuments like the one in Berlin (Figure 3) commemorate his transformative contributions. von Graefe's pioneering methods and dedication have solidified his role as a central figure in the evolution of ophthalmology, with his work continuing to inspire and shape the specialty's progress. 

His work remains highly honored, with several papers considered foundational in ophthalmic research [22]. His influential journal, "Graefe's Archive for Ophthalmology," founded in 1854, continues to be a premier platform for cutting-edge research and advancements in the specialty. von Graefe's legacy is preserved through prestigious awards like the Graefe-Preis and the Graefe-Medal, which recognize outstanding contributions to the field. Albrecht von Graefe's impact on the rise of ophthalmology as a distinct medical specialty is profound and enduring.

## Conclusions

Albrecht von Graefe's pioneering work fundamentally transformed ophthalmology from a subset of general surgery into a respected and distinct medical specialty. His innovative contributions, including the development of iridectomy for glaucoma, the introduction of advanced diagnostic tools, and the refinement of cataract surgery techniques, not only improved patient outcomes but also set new standards for the field. By founding the German Ophthalmological Society and "Graefe's Archive for Ophthalmology," von Graefe established institutions that continue to advance the science and practice of eye care. His commitment to both clinical excellence and research laid a solid foundation for modern ophthalmology, ensuring its recognition as a vital area of medical practice. Today, von Graefe's legacy endures through prestigious awards, ongoing research in his journal, and the continued progress of the specialty he helped to define. His contributions remain a touchstone for innovation and excellence in ophthalmology, reflecting his profound and lasting impact on the field.
